# Influence of surface water and groundwater on functional traits and trade-off strategies of oasis communities at the end of the Keriya River, China

**DOI:** 10.3389/fpls.2024.1340137

**Published:** 2024-02-15

**Authors:** Haobo Shi, Qingdong Shi, Xiaolong Zhou, Chuandeng Cui, Xiang Li, Zipeng Zhang, Chuanmei Zhu

**Affiliations:** ^1^ College of Ecology and Environment, Xinjiang University, Urumqi, China; ^2^ Key Laboratory of Oasis Ecology of Education Ministry, Xinjiang University, Urumqi, China; ^3^ College of Geography and Remote Sensing Sciences, Xinjiang University, Urumqi, China

**Keywords:** surface water disturbance, groundwater depth, functional traits, interspecific variability, intraspecific variability

## Abstract

Plant functional traits reflect the capacity of plants to adapt to their environment and the underlying optimization mechanisms. However, few studies have investigated trade-off strategies for functional traits in desert-wetland ecosystems, the mechanisms by which surface water disturbance and groundwater depth drive functional trait variation at the community scale, and the roles of intraspecific and interspecific variation. Therefore, this study analyzed specific differences in community-weighted mean traits among habitat types and obtained the relative contribution of intraspecific and interspecific variation by decomposing community-weighted mean traits, focusing on the Daliyabuyi Oasis in the hinterland of the Taklamakan Desert. We also explored the mechanisms by which surface water and groundwater influence different sources of variability specifically. The results showed that plant height, relative chlorophyll content, leaf thickness, leaf nitrogen content, and nitrogen-phosphorus ratio were the key traits reflecting habitat differences. As the groundwater depth becomes shallower and surface water disturbance intensifies, plant communities tend to have higher leaf nitrogen content, nitrogen-phosphorus ratio, and relative chlorophyll content and lower height. Surface water, groundwater, soil water content, and total soil nitrogen can influence interspecific and intraspecific variation in these traits through direct and indirect effects. As arid to wet habitats change, plant trade-off strategies for resources will shift from conservative to acquisitive. The study concluded that community functional traits are mainly contributed by interspecific variation, but consideration of intraspecific variation and the covariation effects that exist between it and interspecific variation can help to further enhance the understanding of the response of community traits in desert-wetland ecosystems to environmental change. Surface water disturbance has a non-negligible contribution to this adaptation process and plays a higher role than groundwater depth.

## 1 Introduction

Plant functional traits are core attributes that reflect the response of vegetation to environmental change and strongly influence ecosystem function (Huxley et al., 2023; Jiang et al., 2023). Studies aimed at elucidating the formation mechanisms of functional traits enable the exploration of community ecology-related issues. Functional traits can be studied on multiple scales, and research results have been accumulated at individual as well as ecosystem levels (Roscher et al., 2018; Li et al., 2021; Ouyang et al., 2023). Since the introduction of the leaf economics spectrum concept in 2004, research focused on single traits or groups of traits has gradually shifted to strategies involving trade-offs among traits (Wright et al., 2004). The two ends of the leaf economics spectrum represent the conservative strategy (typically a longer leaf longevity and a lower specific leaf area, leaf nitrogen content, and photosynthetic rate) and the acquisitive strategy (typically a shorter leaf longevity and a higher specific leaf area, leaf nitrogen content, and photosynthetic rate) (Pan et al., 2020). Gathering information on the trade-off characteristics of traits enables researchers to interpret the adaptive strategies of plants under different environmental conditions and helps understanding the mechanisms underlying coexistence between species.

The response-effect trait theory suggests that species’ responses to the environment and their impacts on ecosystem functions are mediated by functional traits (Suding et al., 2008). The community-weighted mean (CWM) trait allows the mean value of traits to be calculated at the community scale using the values of community characteristics as weights (Garnier et al., 2004). The method takes the growth statuses of dominant and non-dominant species in the community into consideration and effectively identifies trait differences between communities. The trade-off strategies of traits illustrate the process of variation that exists in their response to environmental change. This variation can be categorized as inter- or intraspecific. In general, interspecific variability refers to changes in environmental conditions that cause changes in community species composition, known as species turnover (Hogan et al., 2023). In contrast, intraspecific variability refers to environmental effects on phenotypic plasticity in the same species that lead to changes in traits (Siefert et al., 2015).

The degree of intraspecific variation varies by species and trait but is associated with spatial and temporal heterogeneity in environmental conditions (Westerband et al., 2021; Wu et al., 2021). Researchers have begun to gradually focus on intraspecific variation in functional traits over the last decade and have launched a series of studies based on environmental gradients. Kichenin et al. (2013) quantified interspecific versus intraspecific variation in community functional traits across an altitudinal gradient and concluded that positive versus negative covariation between the two reveals plant responses to environmental change; Tusifujiang et al. (2021) concluded that desert plant adaptation decreases gradually when the environment changes from saline to drought stress based on the decrease in intraspecific variation due to increased drought stress. Many previous studies have tended to focus only on the effects exerted by interspecific variability on CWM traits, thereby unconsciously weakening the understanding of adaptive capacity and variability of the species themselves. Therefore, quantifying the relative contributions made by interspecific and intraspecific variability and analyzing their formation process may lead to a better understanding of the driving mechanisms of traits at the community scale (Paź-Dyderska et al., 2020; Xu et al., 2020).

Desert-wetland ecosystems are common in inland river basins in arid regions. Affected by drought and seasonal floods, there are significant differences in the habitats within the ecosystem. Desert-wetland ecosystems sustain a wider range of species and are important sites for understanding local biogeographic history and ecological adaptations of plants (Fensham et al., 2011; Galal et al., 2021). With the intensification of global warming, many regions, including Northwest China, are experiencing dramatic fluctuations in severe droughts and destructive flood events, resulting in huge impacts on the ecological environment (Qing et al., 2023). For Northwest China, increased glacier melt and flooding events have led to a “warming and humidification” trend, especially in the inland river basins of the arid zone (Yang et al., 2021; Chen et al., 2023). Desert-wetland ecosystems are areas of high incidence of these hydrological events. However, the mechanisms driving plant functional trait differences and intraspecific and interspecific variation in this ecosystem are still unknown, which is extremely detrimental to the understanding of plant response patterns and trade-off strategies for future extreme hydrological events.

This study selected the natural oasis at the end of the Keriya River, which is a typical desert-wetland ecosystem, for study. We divided functional traits at the community scale into two components, interspecific and intraspecific variation, and assessed the characteristics of functional traits in response to environmental factors such as surface water disturbance and groundwater depth. We hypothesized that (i) surface water and groundwater will influence intraspecific and interspecific variation in community functional traits and shape existing functional trait characteristics through covariation effects; (ii) The contribution of interspecific variation will be higher than intraspecific variation because species turnover is widespread among habitats; (iii) As habitat types change, trade-off strategies for functional traits will shift.

## 2 Materials and methods

### 2.1 Study area

**Figure 1:**
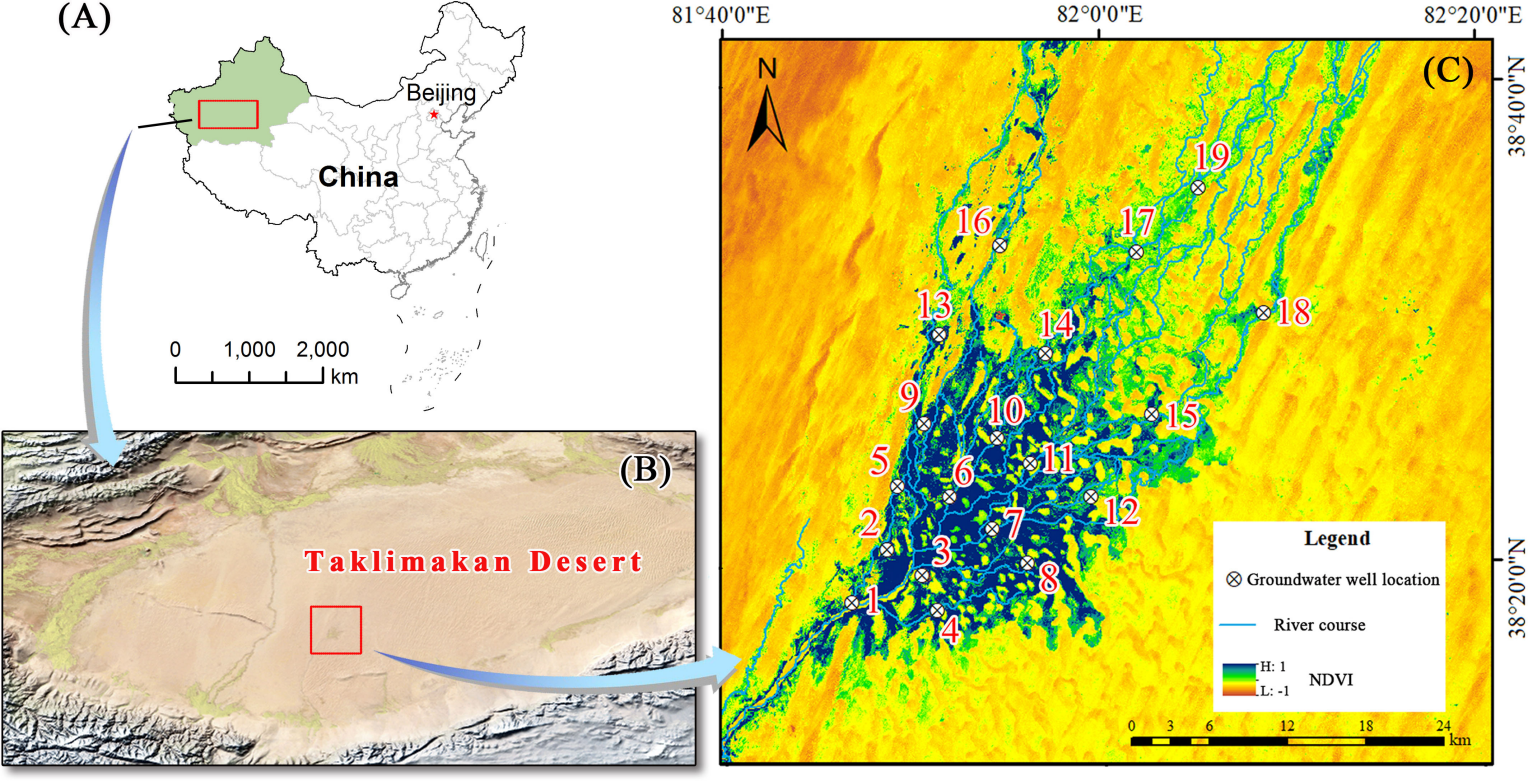
Overview of the study area. **(A)** China map; **(B)** satellite image of the Taklimakan Desert; **(C)** groundwater monitoring wells and plot locations.

The study area was located in the Daliyabuyi Oasis at the end of the Keriya River in Xinjiang, China (38°16′–38°37′ N, 81°41′–82°20′ E). The oasis is in the hinterland of the Taklimakan Desert, with an altitude of 1,100 to 1,300 m and total area of about 342 km^2^. The oasis has a warm-temperate arid desert climate, with an average annual precipitation of less than 20 mm, a potential evaporation of more than 2,000 mm, and a large diurnal temperature range (Peng et al., 2022). Due to scouring by the Keriya River, the internal river channels of the oasis have become complex and depositional characteristics of anastomosing river. However, except for the summer floods, the seasons and timing of flood events are not fixed. During the flood period, some areas experience different degrees of surface water overflow, leading to surface water disturbances on a certain scale (Shi et al., 2022). The plant community of the oasis consists mainly of *Populus euphratica*, *Tamarix chinensis*, *Phragmites australis*, and others. The research team laid several vegetation monitoring transects in the oasis at the end of 2018 and constructed 19 groundwater monitoring wells within these transects, thus acquiring groundwater depth data and establishing a good facility basis for community ecology research (**Figure 1**).

### 2.2 Vegetation survey and functional trait data collection

In the summer of 2021, the research team set up a 50 × 50 m plot near each groundwater monitoring well at the Daliyabuyi Oasis. Each plot was divided into four 25 × 25 m subplots (Zhao and Xu, 2019; Zhang et al., 2022), totaling 76 subplots. As this study focused on the community scale rather than the population scale, subplots with only one species were removed, leaving 71 subplots for the subsequent analyses (Zhao et al., 2022). The plant composition within the subplots was recorded and the number of individuals, height, crown length, and width data were determined separately for all plants (crown length and width data can be used to calculate vegetation coverage for different species). When height (H) is used as a functional trait, it refers to the average height of all individuals of each species within the subplot. A handheld GPS (G120BD, UniStrong, China) was used to locate plots and record information pertaining to geographic coordinates.

First, 8–10 plants of each species at different age stages were selected within each subplot (25 × 25 m) and young and old leaves were evenly collected on each plant. A total of 20–40 leaves were collected for each species within the subplot (Chen et al., 2021). Relative chlorophyll content (SPAD) was determined using a chlorophyll detector (SPAD-502, Konica Minolta Sensing Inc., Japan) as early as possible following leaf collection (Maharjan et al., 2021). Since the measurement of chlorophyll content was shown as SPAD value, the abbreviation of this trait was named as SPAD. Leaves were brought to the laboratory and placed in water for one hour for rehydration. The leaf thickness (LT) was measured using vernier calipers with an accuracy of 0.01 mm. The SPAD and LT of each species in the subplot are the average values calculated after measuring each leaf. Leaves of the same species within each subplot were pooled together and the fresh weight of the leaves was measured using an electronic balance with an accuracy of 0.001 g. Subsequently, the leaves were placed on a square mesh paper and covered with a transparent glass plate. The cleaned leaves were scanned at a resolution of 300 dpi using a portable scanner (Epson, V19, Japan) and the leaf area was extracted using Adobe Photoshop CS6 software. After drying in an oven at 75°C for 48 hours, the dry weight, specific leaf area (SLA), and leaf dry matter content (LDMC) were determined. Next, the dried leaves of the same species from each subplot were thoroughly mixed, crushed and sieved (0.15 mm) and the carbon (LCC), nitrogen (LNC), and phosphorus (LPC) contents of the leaves were measured. LCC and LNC were determined using an elemental analyzer (Vario EL cube, Elementar, Germany) and LPC was determined using the molybdenum antimony anti-colorimetric method (Ling-Ling et al., 2023). The ratios between LCC, LNC, and LPC were calculated to obtain C/N, C/P, and N/P.

### 2.3 Quantification of environmental factors

Three 100 cm deep soil profiles were randomly set up within each plot, and each profile was divided into six layers (0–5, 5–20, 20–40, 40–60, 60–80, and 80–100 cm) for sample collection purposes. The same layer of soil from three locations was thoroughly mixed after collection, at which point the six mixed soil samples represented soil conditions at different depths at the community level. The mixed soil samples were brought back to the laboratory in aluminum boxes and plastic bags, respectively, and soil water content, total dissolved solids, pH, organic matter, total nitrogen, and total phosphorus were determined. Soil water content was measured using the drying method; total dissolved solids and pH were determined using a conductivity meter (DDSJ-319L, Rex, China) and pH meter (PHSJ-5T, Rex, China), respectively; organic matter was determined using the potassium dichromate external heating method (Yuan et al., 2023); and total nitrogen and total phosphorus were determined in the same way as for leaves. Mean values of full profiles were used for each soil variable for subsequent analysis.

**Figure 2:**
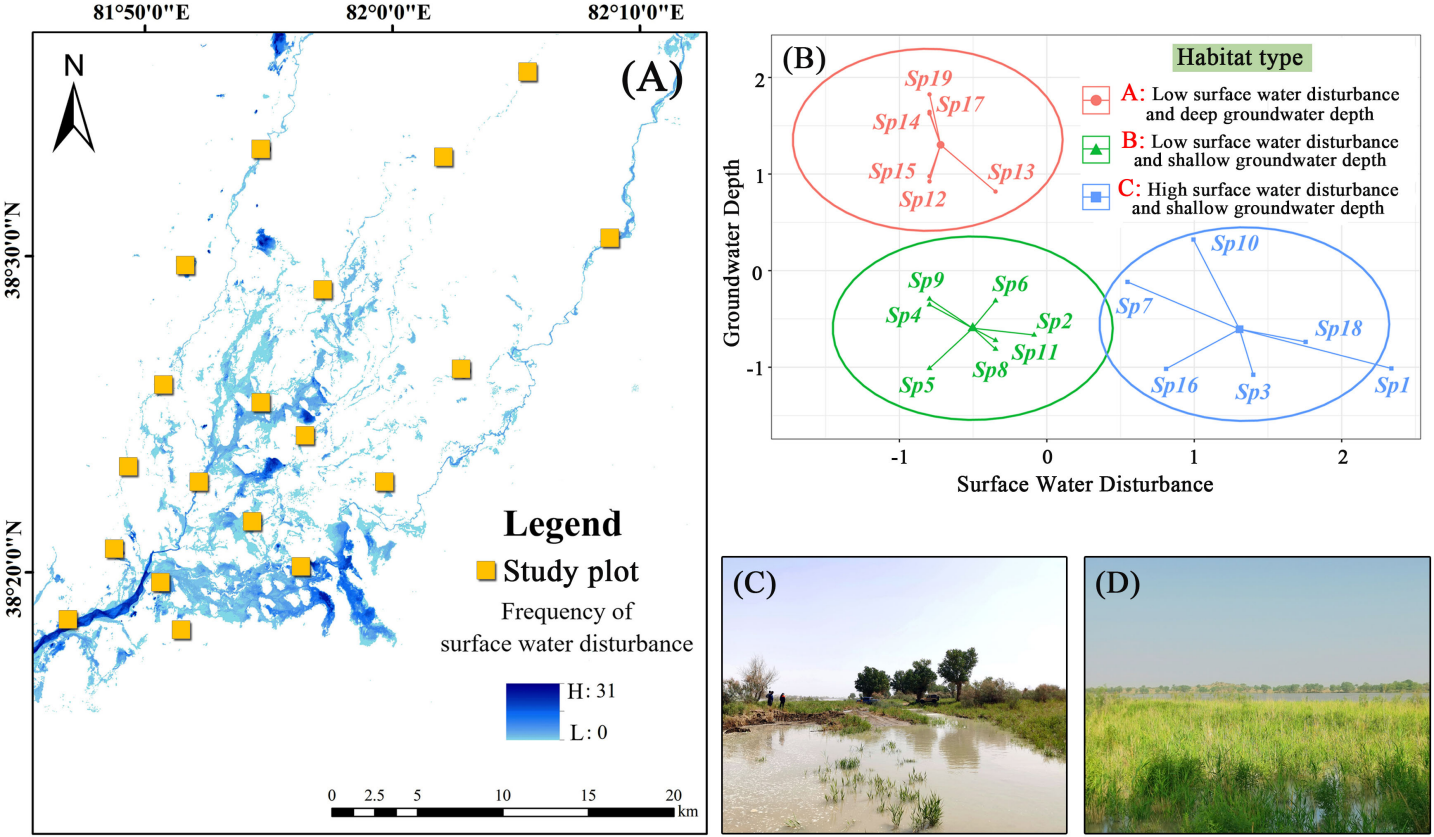
K-mean clustering results of surface water disturbance and groundwater depth. **(A)** frequency of surface water distribution; **(B)** K-mean clustering; **(C, D)** examples of surface water disturbance.


We obtained groundwater depth data recorded by groundwater monitoring wells for the plant growing season (April to October) from 2019 to 2021 and calculated the average value for subsequent analysis. Flood-related damage to the monitoring well in plot 16 resulted in missing data, due to which groundwater depth in this plot was calculated using data from 2019 and 2020 only. Selected Landsat 8 remote sensing images from 2017 to 2021 were used to obtain the frequency characteristics of surface water distribution via extraction of the water index and threshold segmentation techniques, which were validated via several field observations (**Figure 2A**). The extent of surface water disturbance to the community was quantified using the following formula:

$$\;\text{SWD}=\text{ln}\sum_{\text{i}=1}^{\text{S}}{\text{w}}_{\text{i}}$$

where S represents the number of surface water raster cells within 1 ha of the positioning point and w_i_ represents the frequency of the ith raster cell in the monitoring time frame. SWD represents the degree of surface water interference at the community scale after considering the frequency and total amount of overflow. The obtained surface water disturbance and groundwater depth of data were standardized, and the habitat types were classified using the K-means clustering method.

### 2.4 Data analysis

Species importance values were used as weights to calculate CWM traits (H, SPAD, LT, SLA, LDMC, LCC, LNC, LPC, C/N, C/P, N/P) via the following method:


$$\text{CWM}\;\text{trait}=\sum_{\text{j}=1}^{\text{n}}({\text{IV}}_{\text{j}}\times {\text{trait}}_{\text{j}})$$

$$\text{I}{\text{V}}_{\text{j}}=\left({\text{Ar}}_{\text{j}}+{\text{Hr}}_{\text{j}}+{\text{Cr}}_{\text{j}}\right)/3$$

where IV_j_ and trait_j_ represent the importance value and average trait value of species j in each subplot, respectively, Ar_j_ denotes relative number of individuals, Hr_j_ denotes relative height, and Cr_j_ denotes relative coverage. The calculation process involved the use of “FDiversity” software developed in R language (Casanoves et al., 2011).

Normality and homogeneity of variance tests were performed for each CWM trait in the study area. Data satisfying both normal distribution and homogeneity of variance were analyzed using the least significance difference (LSD) method to compare differences between habitat types; data that satisfied the normal distribution but did not pass the homogeneity of variance test were analyzed using Tamhane’s T2 method; data that did not satisfy the normal distribution were compared using the Kruskal-Wallis test. The surface water disturbance and groundwater depth were used as independent variables and CWM traits, which differed significantly among habitat types, were used as the dependent variables. The relationships between the independent and dependent variables were analyzed using simple linear regression. The above data were standardized before linear regression. To determine the trade-offs between functional traits, we also performed factor analyses on the trait data after standardization. The Varimax rotation method was used to reduce the correlation between factors to obtain a factor structure that is easy to interpret.

Leps’ method was used to calculate inter- and intraspecific variability in community traits as follows:


$$\;\mathrm{interspecific}\;\mathrm{variability}=\sum_{\text{j}=1}^{\text{n}}({\text{IV}}_{\text{j}}\times {\text{trait}}_{\text{j}\_\text{mean}})$$

intraspecific variability = CWM trait–interspecific variability

where trait_j_mean_ refers to the fixed average trait value of species j, that is, the average of traits of species j in all subplots. Differences in fixed mean trait values between communities are generally considered to be caused by species turnover, whereas differences between CWM trait values and fixed mean trait values are considered to be due to intraspecific trait variation only. Thus, we obtained two new parameters of interspecific and intraspecific variability via this method. In addition to being able to use them as new response variables, it is also possible to obtain the relative contribution of these two parameters to the CWM traits based on the sum-of-squares decomposition (Lepš et al., 2011).

The study also used systematic clustering and two-way analysis of variance (ANOVA) to analyze the relative contribution and significance of surface water and groundwater to interspecific and intraspecific variability. Systematic clustering can categorize surface water disturbance and groundwater depth into different levels each, and both become categorical variables. The above analyses were conducted using the “cluster” package (Maechler et al., 2022) and the “trait.flex.nova” function in R 4.2.3. Finally, the mediating effects of soil physicochemical properties were analyzed using structural equation modeling (SEM) to clarify the direct and indirect effects of surface water disturbance and groundwater depth on the two components of CWM traits. The model construction process screened the soil factors according to the multicollinearity and selected the appropriate model according to the Akaike Information Criterion. This process used the “piecewiseSEM” package (Lefcheck, 2016).

## 3 Results

### 3.1 Differences in CWM traits among habitat types

**Figure 3:**
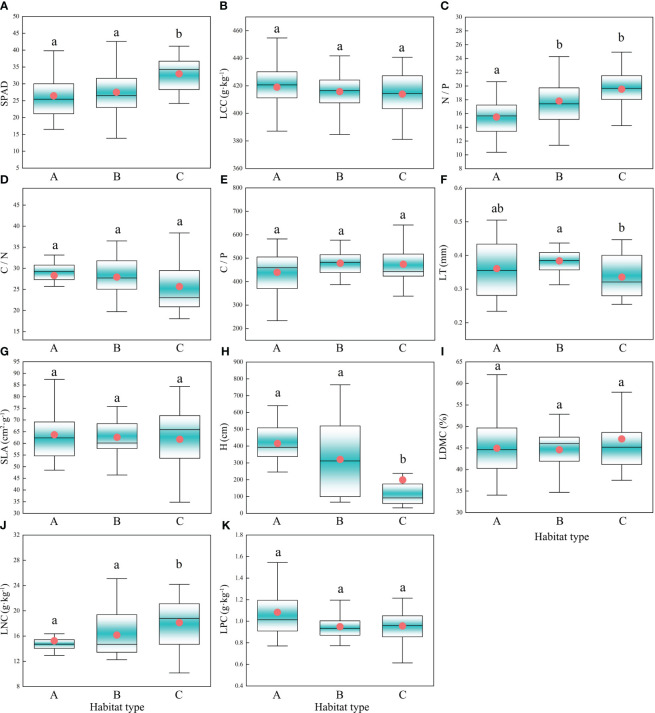
Characteristics of CWM trait changes under different habitat types. Different letters indicate significant differences (P<0.05) between habitat types; The circles in the box plots represent the average value and the horizontal lines in the middle represent the median; Habitat type A, Low surface water disturbance and deep groundwater depth; Habitat type B, Low surface water disturbance and shallow groundwater depth; Habitat type C, High surface water disturbance and shallow groundwater depth; **(A)** SPAD, Relative chlorophyll content; **(B)** LCC, Leaf carbon content; **(C)** N/P, Nitrogen to phosphorus ratio; **(D)** C/N, Carbon to nitrogen ratio; **(E)** C/P, Carbon to phosphorus ratio; **(F)** LT, Leaf thickness; **(G)** SLA, Specific leaf area; **(H)** H, Height; **(I)** LDMC, Leaf dry matter content; **(J)** LNC, Leaf nitrogen content; **(K)** LPC, Leaf phosphorus content.

K-means clustering can divide the study area’s habitats into three types (**Figure 2B**), namely low surface water disturbance and deep groundwater depth (habitat type A), low surface water disturbance and shallow groundwater depth (habitat type B), and high surface water disturbance and shallow groundwater depth (habitat type C). The results showed that five CWM traits, including the SPAD, N/P, LT, H, and LNC, showed significant differences among different habitat types. The highest SPAD was observed for habitat type C, which was significantly higher than those observed for the other two habitat types (**Figure 3A**). N/P was lowest in habitat type A, with a mean value of 15.49, significantly lower than those observed for the other two habitat types (**Figure 3C**). LT was highest in habitat type B, which was significantly higher than that of habitat type C (**Figure 3F**). H was lowest for habitat type C (median value of 92.07), which was significantly lower than those observed for habitat types A and B (**Figure 3H**). In habitat type C, the median LNC (18.78) was significantly higher than those of the other two habitat types (**Figure 3J**).

### 3.2 Response of CWM traits to surface water and groundwater

**Figure 4:**
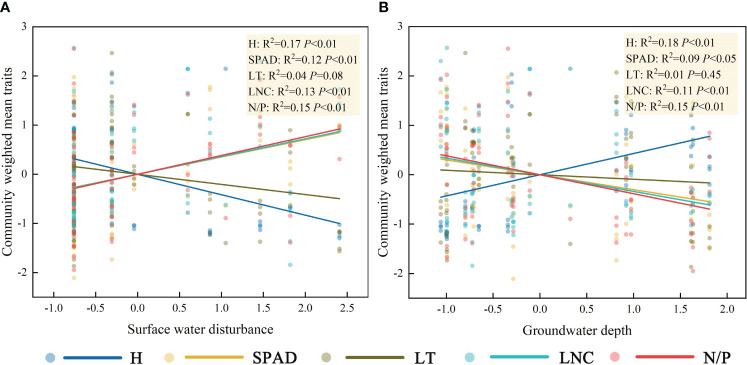
Characterization of the linear response of community functional traits to surface water disturbance **(A)** and groundwater depth **(B)**. The abbreviations for functional traits are the same as in **Figure 3**.

Linear regression results showed a significant negative correlation between H and surface water disturbance and significant positive correlations between SPAD, LNC, N/P, and surface water disturbance (**Figure 4A**). There was a significant positive correlation between H and groundwater depth and significant negative correlations between SPAD, LNC, N/P, and groundwater depth (**Figure 4B**). LT did not show a clear pattern of response to either surface water disturbance or groundwater depth (*P*>0.05). It is noteworthy that despite the general linear relationship observed between surface water disturbance, groundwater depth, and CWM traits, the goodness of fit for all linear regressions remains low.

### 3.3 Trade-off strategies for community functional traits

**Figure 5:**
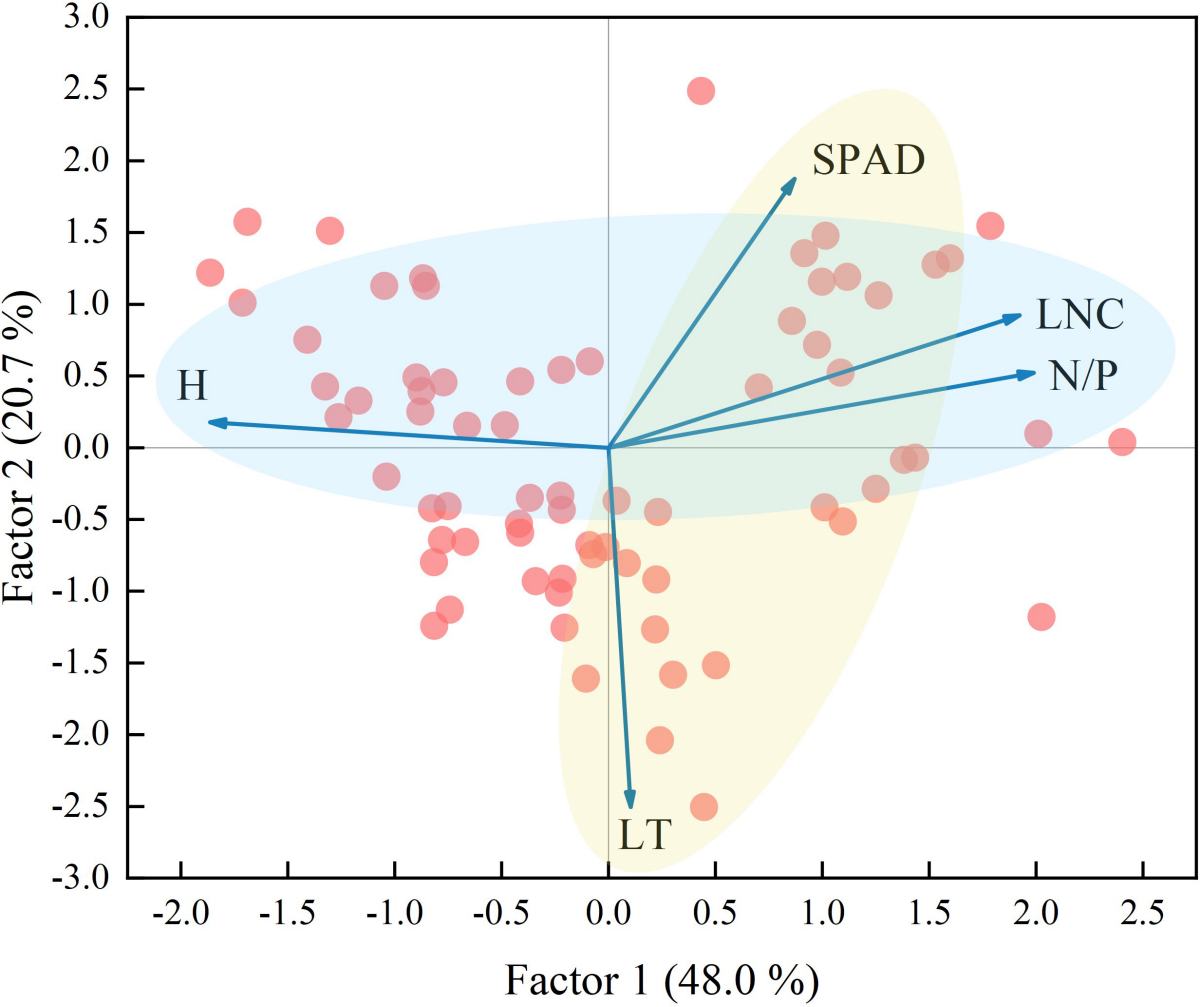
Factor analysis of community weighted mean traits. The light blue circle represents Factor 1 and the light yellow circle represents Factor 2. The abbreviations for functional traits are the same as in **Figure 3**.

Factor analysis was performed on the five functional traits, and two factors were extracted. The variance explained by the factors, which were 48.0% and 20.7%, respectively, represented 68.7% of the information content of the original data (**Figure 5**). The use of factor loading coefficients derived from factor rotations as classification criteria resulted in H, N/P, and LNC being included in Factor 1, and SPAD, LT in Factor 2 (**Supplementary Table 4**). The traits in Factor 1 represented plant growth and nutrient storage status, while the traits in Factor 2 were closely related to the photosynthetic capacity of the plant. There were negative correlations between H and LNC and N/P and positive correlations between LNC and N/P (Factor 1). There was a negative correlation between SPAD and LT (Factor 2). According to **Figures 3** and **4** and the leaf economics spectrum theory, as groundwater becomes shallower and the degree of surface water disturbance increases, functional traits at the community scale would change from a conservative strategy of “slow investment-return” to an acquisitive strategy of “quick investment-return”. This result accepts the third hypothesis that trade-off strategies for functional traits change as habitat type changes.

### 3.4 Sources of variation in functional traits

**Figure 6:**
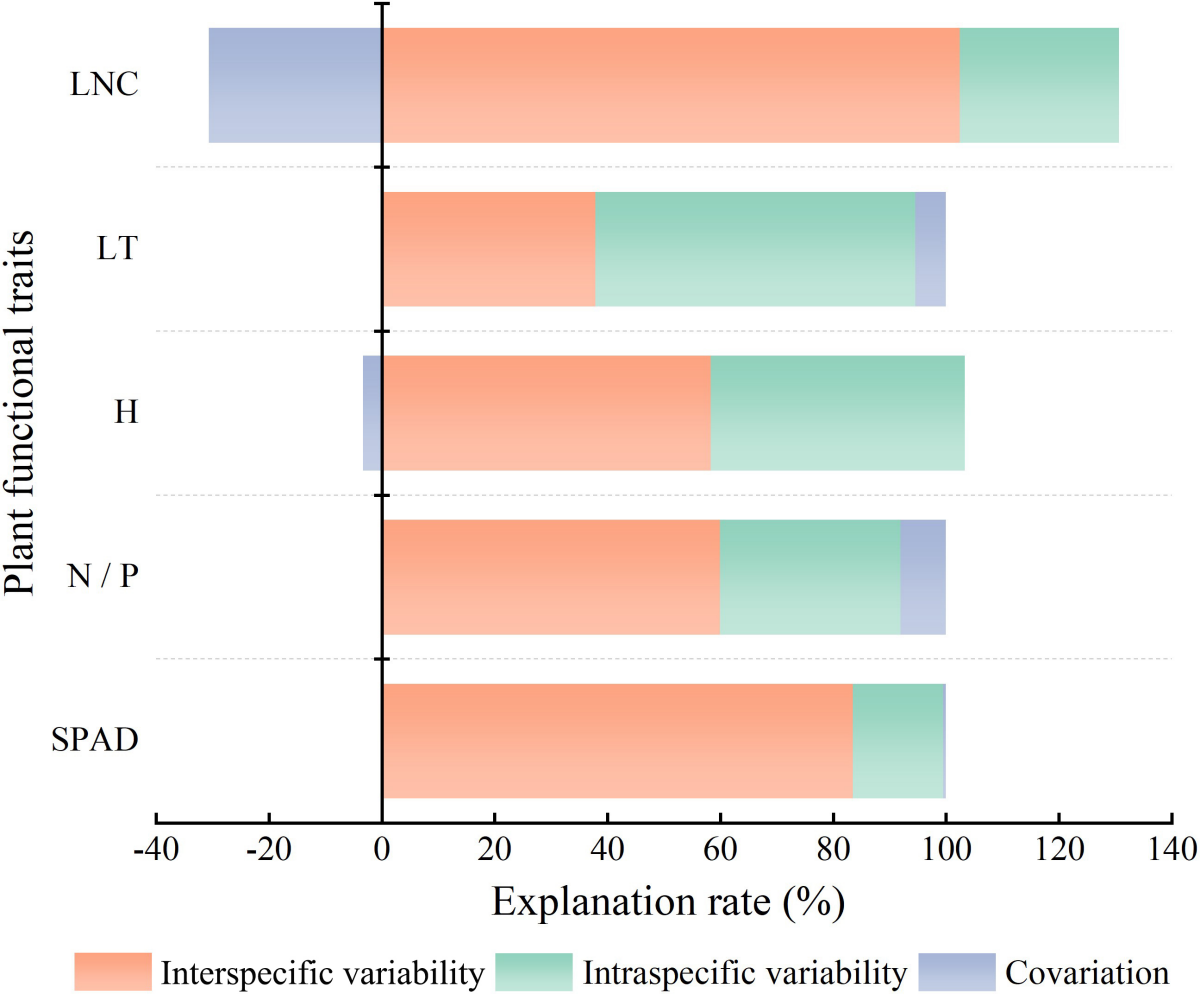
Source decomposition of CWM trait variation. The abbreviations for functional traits are the same as in **Figure 3**. All traits are community-weighted mean traits.

The contribution of interspecific versus intraspecific variation (**Figure 6**) revealed that the total variation in LNC was mainly derived from interspecific variability, which was 3.62 times higher than intraspecific variability. The proportions of interspecific and intraspecific variability for H were nearly identical, at 58.3% and 45.1%, respectively. The variation in SPAD, which was mainly derived from interspecific variability, accounted for 83.5% of the total variation. The proportion of interspecific variability (59.9%) in N/P was higher than that of intraspecific variability (32.0%). Of the total variation in LT, the proportion of interspecific variability (37.9%) was lower than that of intraspecific variability (56.7%). Interspecific and intraspecific variability showed positive covariation in the effects on LT, N/P, and SPAD and negative covariation in the effects on LNC and H. Combined with the linear regression results in **Figure 4**, this indicated that as community H gradually decreased, the H of dominant species showed a weak increasing trend, and as community LNC gradually increased, the LNC of the dominant species decreased.

**Figure 7:**
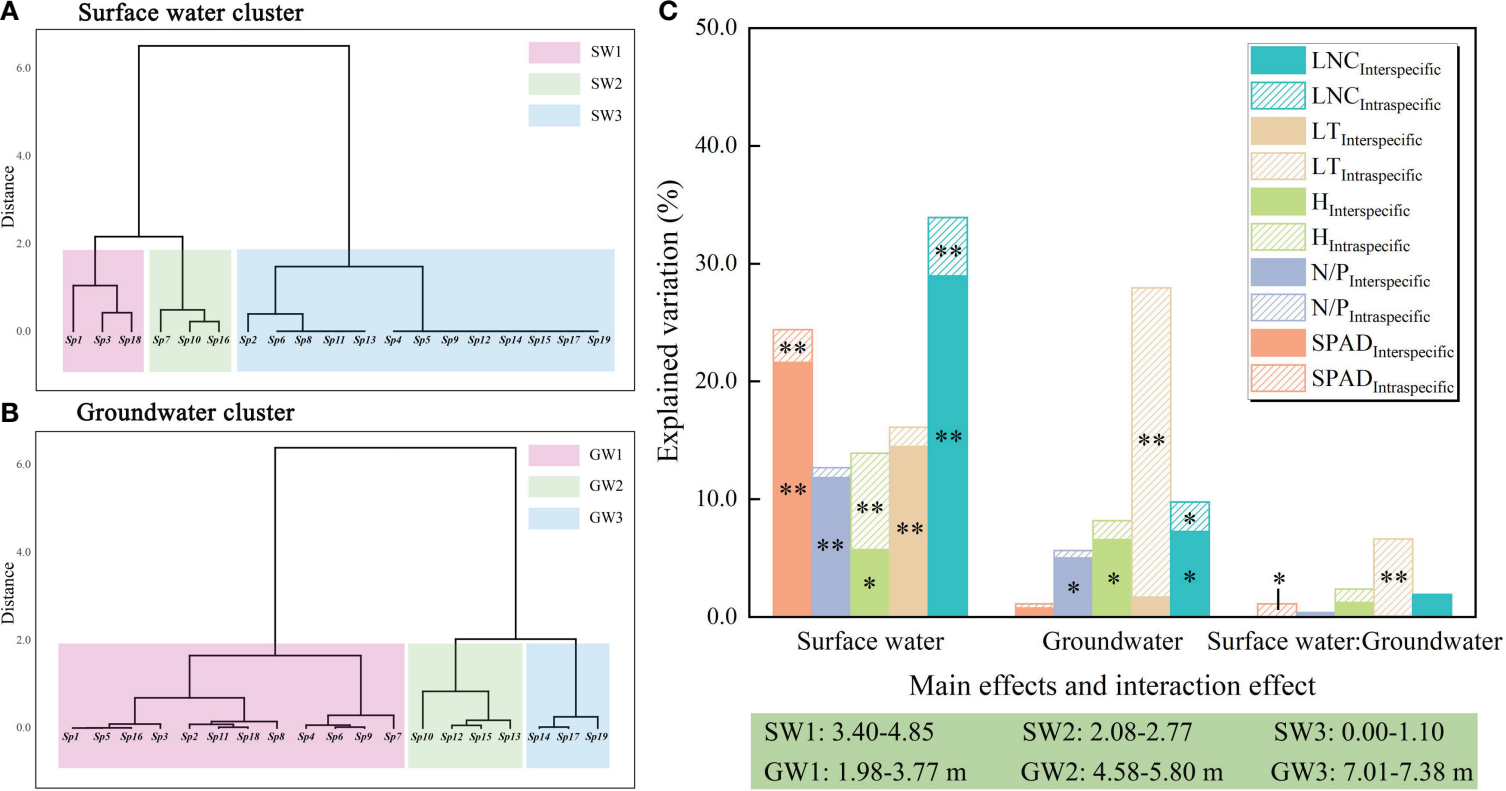
Two-way ANOVA and variance decomposition results for surface water disturbance and groundwater depth. **: *P*<0.01; *: *P*<0.05; **(A, B)** results of systematic clustering of surface water disturbance and groundwater depth; **(C)** two-way ANOVA. The abbreviations for functional traits are the same as in **Figure 3**.

Two-way ANOVA indicated that surface water disturbance exerted a significant effect on the variation in community traits due to interspecific variability, with explanatory rates of 21.6% (SPAD), 11.8% (N/P), 5.7% (H), 14.5% (LT), and 29.0% (LNC), respectively. Surface water disturbance also exerted a significant effect on the intraspecific variability of the two traits, SPAD and LNC, which showed explanatory rates of 2.8% and 5.0%, respectively. Groundwater depth exerted a significant effect on the interspecific variability components, N/P, H, and LNC, explaining 5.0%, 6.6%, and 7.3%, respectively. Groundwater depth also had a significant effect on intraspecific variability in LT and LNC, with explanatory rates of 26.2% and 2.5%, respectively. The interaction between surface water and groundwater exerted a significant effect on the intraspecific variability of SPAD and LT, with explanation rates of 1.1% and 6.5%, respectively (**Figure 7**).

### 3.5 Effects of surface water, groundwater, and soil factors on CWM traits

**Figure 8:**
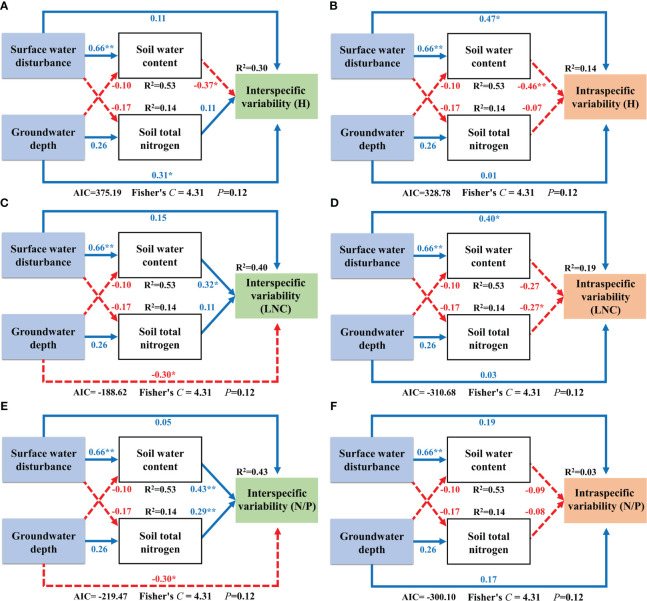
Structural equation modeling of surface water and groundwater effects on Factor 1. **: *P*<0.01; *: *P*<0.05; The blue solid line in the figure represents positive impact, and the red dotted line represents negative impact; The abbreviations for functional traits are the same as in **Figure 3**; **(A, B)** interspecific variability and intraspecific variability of height; **(C, D)** interspecific variability and intraspecific variability of leaf nitrogen content; **(E, F)** interspecific variability and intraspecific variability of nitrogen to phosphorus ratio.

We analyzed the driving mechanisms of interspecific and intraspecific variability by constructing SEMs that incorporated soil factors as an intermediate medium. Surface water disturbance exerted a significantly positive effect on soil water content. The SEM results for Factor 1 showed that interspecific variability of H was directly and positively influenced by groundwater depth and an indirect negative effect on it by surface water acting on soil water content (**Figure 8A**). Surface water disturbance exerted a direct positive effect on the intraspecific variability of H and an indirect negative effect on it by acting on soil water content (**Figure 8B**). The interspecific variability component in LNC was directly negatively influenced by groundwater depth and indirectly positively influenced by surface water via soil water content (**Figure 8C**). The intraspecific variability component of LNC was directly positively and negatively influenced by surface water disturbance and soil total nitrogen, respectively (**Figure 8D**). The interspecific variability component of N/P was directly and negatively influenced by the groundwater depth and indirectly positively influenced by surface water via soil water content. In addition, soil total nitrogen exerted a direct positive effect on the interspecific variability of N/P (**Figure 8E**). For the intraspecific variability component of N/P, no direct or indirect effects of surface water and groundwater were found (**Figure 7**; **Figure 8F**).

**Figure 9:**
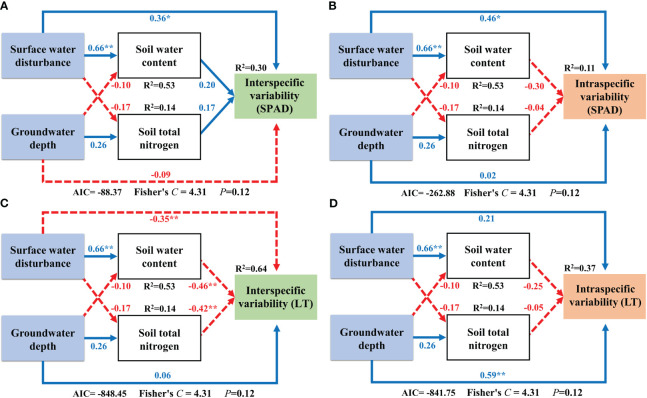
Structural equation modeling of surface water and groundwater effects on Factor 2. **: *P*<0.01; *: *P*<0.05; The blue solid line in the figure represents positive impact, and the red dotted line represents negative impact; The abbreviations for functional traits are the same as in **Figure 3**; **(A, B)** interspecific variability and intraspecific variability of relative chlorophyll content; **(C, D)** interspecific variability and intraspecific variability of leaf thickness.

The SEM results for Factor 2 showed that both interspecific and intraspecific variability components of SPAD were directly and positively affected by surface water disturbance and that no other effects were present (**Figure 9A**; **Figure 9B**). Surface water disturbance exerted a direct negative effect on the interspecific variability of LT, as well as an indirect negative effect via the regulation of soil water content. Soil total nitrogen also exerted a direct negative effect on interspecific variability in LT (**Figure 9C**). The intraspecific variability of LT was only directly and positively influenced by groundwater depth (**Figure 9D**).

## 4 Discussion

### 4.1 Effects of habitat heterogeneity on functional traits

The habitat type shifted from A to B as the groundwater depth shifted from deep to shallow. Considering the strong dependence of desert plants on groundwater, such shifting enables most species to fulfill their water needs (Glanville et al., 2023). At this time, both traits of SPAD and LNC showed a weak upward trend at the community level, indicating that the photosynthetic rate was also slightly increased (Mu and Chen, 2021; Wang et al., 2021), even though the above trends did not constitute a significant difference. N/P showed a significant increase after groundwater depth became shallower, with the community showing some degree of phosphorus limitation (Koerselman and Meuleman, 1996; Cao and Chen, 2017). Zhang et al. (2018) concluded that shallower groundwater depths reduce soil phosphorus levels, a trend also found in this study (**Supplementary Figure 1**), which may be the underlying cause of phosphorus limitation. Thicker leaves imply longer leaf longevity (Pérez-Harguindeguy et al., 2016), and this study suggests that relying on differences in groundwater depth alone is not sufficient to change the leaf longevity profile of the community. Chen et al. (2021) found that groundwater depth resulted in changes in the LT of trees and shrubs, but there was no clear pattern of effect on the LT of herbaceous plants. Thus, the response of LT to groundwater depth may depend on plant life form.

SPAD was significantly enhanced after surface water disturbance increased (habitat type C). The significant decrease in LT enhanced the ability of leaves to intercept light, indicating a decline in leaf longevity in the community. The significant decrease in H caused by shallower groundwater depth and increased surface water disturbance may be attributed to the improved water environment which allows more herbaceous species to emerge, while the overlapping ecological niches may have also caused species, such as *P. euphratica*, to respond to resource competition by reducing their dominance (Shi et al., 2021). It has been noted that excessive surface water disturbance may cause waterlogging that increases soil salinity, thereby severely inhibiting the photosynthesis and growth of *P. euphratica* (Ma et al., 1997). Previous studies have suggested a positive correlation between LNC and photosynthetic- as well as respiration- rates (Evans, 1989; Dalke et al., 2018). The significant positive correlation between LNC and SPAD in this study verified the above statement (**Supplementary Figure 2**), while the lack of significant differences in LCC between habitat types also side-stepped the existence of a positive correlation between photosynthetic and respiration rates (Faber et al., 2022).

### 4.2 Role of environmental factors in the formation of CWM traits

Although the linear regressions between surface water, groundwater, and CWM traits in this study had statistical significance, the R^2^ values were low, indicating that other factors were involved in influencing functional traits. On the one hand, this study only considered the aboveground traits of plants, but the belowground traits are likely to have a more pronounced response pattern to water resource differences (Klimešová et al., 2023). On the other hand, competition between species may also be an important factor affecting trait plasticity, especially in regions with scarce resources (Berg and Ellers, 2010). Govaert et al. (2021) pointed out in their study that the traits of species depend not only on the degree of environmental change, but also on interspecies interactions.

The effects of soil moisture and salinity on the functional traits of communities in arid zones have been revealed by several studies (Gong et al., 2019; Luo et al., 2021). However, in the present study, soil factors were observed to assume a more mediatory role. During the screening of variables for structural equation model construction, it was found that the Akaike Information Criterion values were much higher than those of the current model when soil water content and salinity were included in the model, although the null hypothesis was not rejected (*P*>0.05). This may be due to both LNC and N/P being correlated with nitrogen content, indicating that the role of soil total nitrogen on interspecific and intraspecific variability may be closer to the theoretical model than soil salinity. The model showed that total soil nitrogen did not show a significant response pattern to surface water disturbance and groundwater depth, which is consistent with the findings of some studies (Zhang et al., 2018; Chen et al., 2020). However, the conclusion should consider the differences in time scales with other studies. When it is difficult to precisely capture the timing of the occurrence of surface runoff and underground leaching during the study process, their specific contribution to soil total nitrogen cannot be denied (Zhang et al., 2020; Bao et al., 2023) but can only indicate the existence of stability of soil total nitrogen under a given ecosystem. Due to the complexity of the spatial variation of environmental factors, the adaptive mechanism of plant communities to the environment is still uncertain and it is necessary to use fixed sample plots and continuous monitoring methods to conduct in-depth research.

### 4.3 Interspecific and intraspecific variability combine to drive changes in functional traits in oasis communities

In this study, the proportions of interspecific variability of SPAD, H, LNC, and N/P were higher than those of intraspecific variability. Changes in water environmental conditions in desert-wetland complex ecosystems may introduce new trait values that improve the adaptive capacity of the whole community. If the distance within the environmental gradient is too large, it may lead to an increase in the importance of species turnover (Siefert et al., 2014). However, the results of the study showed that the proportion of intraspecific variability was not much lower than that of interspecific variability, which could be due to both surface water and groundwater gradients being set appropriately and the capacity for potential phenotypic plasticity of the species being depleted by environmental changes (Auger and Shipley, 2013). Regardless of the cause, the importance of intraspecific variability in community trait variation cannot be ignored. There were positive covariation effects of intra- and interspecific variation in LT, SPAD, and N/P. LT has been used as an example to demonstrate that in communities where species with thin leaves are dominant, species will generally grow thinner leaves than expected. There was a negative covariation effect of intra- and interspecific variation of H and LNC, which is generally considered to be a weakening effect (Luo et al., 2023) and a negative compensatory mechanism (Xiang et al., 2021). Taking H as an example, in communities where taller species dominate, the weighted mean height of the community is lower than would otherwise be expected. The negative covariation elaborates the following mechanism: During the transition from arid to wet habitats, the dominance of dwarf plants with higher LNC gradually increases but the intraspecific LNC and H will decrease and increase, respectively. Past studies have also observed this phenomenon (Kichenin et al., 2013; Luo et al., 2016). Overall, this result accepts the first two hypotheses presented in the introduction as the contribution of intraspecific variation was lower than that of interspecific variation. However, in the process of environmental change, intraspecific variation influenced the pattern of community traits.

## 5 Conclusions

As the groundwater depth decreased and surface water disturbance increased, plant communities in desert wetland ecosystems tended to have higher LNC, N/P, and SPAD but lower H. Interspecific and intraspecific variations in traits can be affected directly or indirectly by surface water disturbance, groundwater depth, soil water content, and soil total nitrogen. Although changes in community functional traits are mainly caused by interspecific variation, intraspecific variation also has a non-negligible contribution. In addition, the covariation effect between interspecific and intraspecific variations can explain the formation of existing functional trait characteristics. With the shift from arid to wet habitats, the trade-off strategies of plant traits for resources shifted from conservative to acquisitive. It was concluded that intraspecific variation has been instrumental in enhancing the understanding of functional traits in response to environmental change. Interspecific variation, intraspecific variation, and covariation effects combine to influence community trait characteristics in desert-wetland ecosystems driven by surface water disturbance and groundwater depth, and the role of surface water is higher than that of groundwater in this process. Our study is an important step in unraveling the mechanisms through which surface water and groundwater influence functional traits.

## Data availability statement

The original contributions presented in the study are included in the article/**Supplementary Material**. Further inquiries can be directed to the corresponding author.

## Author contributions

HS: Data curation, Formal analysis, Investigation, Visualization, Writing – original draft. QS: Data curation, Funding acquisition, Project administration, Supervision, Writing – review & editing. XZ: Conceptualization, Methodology, Writing – review & editing. CC: Investigation, Writing – original draft. XL: Investigation, Writing – original draft. ZZ: Methodology, Writing – original draft. CZ: Methodology, Writing – original draft.
