# Supplementary material for: Influence of surface water and groundwater on functional traits and trade-off strategies of oasis communities at the end of the Keriya River, China
Source: Front Plant Sci. 2024 Feb 15;15:1340137. doi: 10.3389/fpls.2024.1340137 (PMC10905963; doi:10.3389/fpls.2024.1340137)
Supplement: Supplementary file 1 [file DataSheet_1.docx]

Supplementary Material

# Supplementary Figures and Tables

## Supplementary Tables

**Supplementary Table 1.** Coordinates of the 19 sample plots

| Sample plot number | Longitude | Latitude |
| --- | --- | --- |
| 1 | 81°46′36.982″ | 38°18′28.690″ |
| 2 | 81°48′29.285″ | 38°20′39.850″ |
| 3 | 81°50′21.871″ | 38°19′35.19″ |
| 4 | 81°51′9.518″ | 38°18′8.026″ |
| 5 | 81°49′04.85″ | 38°23′19.821″ |
| 6 | 81°51′49.615″ | 38°22′50.633″ |
| 7 | 81°54′4.493″ | 38°21′29.577″ |
| 8 | 81°55′57.270″ | 38°20′3.392″ |
| 9 | 81°50′31.014″ | 38°25′54.413″ |
| 10 | 81°54′23.658″ | 38°25′18.111″ |
| 11 | 81°56′4.36″ | 38°24′21.996″ |
| 12 | 81°59′20.854″ | 38°22′49.152″ |
| 13 | 81°51′22.36″ | 38°29′38.24″ |
| 14 | 81°56′59.362″ | 38°28′47.137″ |
| 15 | 82°02′35.345″ | 38°26′13.277″ |
| 16 | 81°54′37.82″ | 38°33′18.52″ |
| 17 | 82°01′52.127″ | 38°32′59.851″ |
| 18 | 82°08′33.557″ | 38°30′21.667″ |
| 19 | 82°05′13.131″ | 38°35′37.73″ |

**Supplementary Table 2.** Community characteristics under different habitat types (mean±SE)

| Habitat type | Number of plant individuals | Sum of the average height of the populations | Vegetation coverage |
| --- | --- | --- | --- |
| A | 48.25±9.50a | 779.25±27.15a | 0.26±0.03 |
| B | 367.71±127.89a | 680.38±74.52a | 0.38±0.04 |
| C | 1122.46±342.10b | 429.41±85.97b | 0.41±0.08 |

Note: Different letters represent significant differences between each other.

**Supplementary Table 3.**  Inventory of species in the study area

| Serial No. | Family | Genus | Species |
| --- | --- | --- | --- |
| 1 | salicaceae | Populus | *Populus euphratica* |
| 2 | tamaricaceae | Tamarix | *Tamarix chinensis* |
| 3 | asteraceae | Karelinia | *Karelinia caspia* |
| 4 | fabaceae | Glycyrrhiza | *Glycyrrhiza uralensis* |
| 5 | poaceae | Phragmites | *Phragmites australis* |
| 6 | fabaceae | Alhagi | *Alhagi sparsifolia* |
| 7 | asteraceae | Hexinia | *Hexinia polydichotoma* |
| 8 | asteraceae | Inula | *Inula salsoloides* |
| 9 | asteraceae | Onopordum | *Onopordum acanthium* |
| 10 | fabaceae | Sophora | *Sophora alopecuroides* |

**Supplementary Table 4.** Factor analysis results (Bartlett's test P<0.01, R2=0.69)

| Functional traits  (CWM) | Factor loading (varimax-rotation method) | | Common factor variance |
| --- | --- | --- | --- |
|  | Factor 1 | Factor 2 |  |
| SPAD | 0.365 | 0.655 | 0.562 |
| LT | 0.044 | -0.875 | 0.767 |
| H | -0.783 | 0.061 | 0.617 |
| LNC | 0.808 | 0.323 | 0.756 |
| N/P | 0.836 | 0.183 | 0.732 |

## Supplementary Figures

**
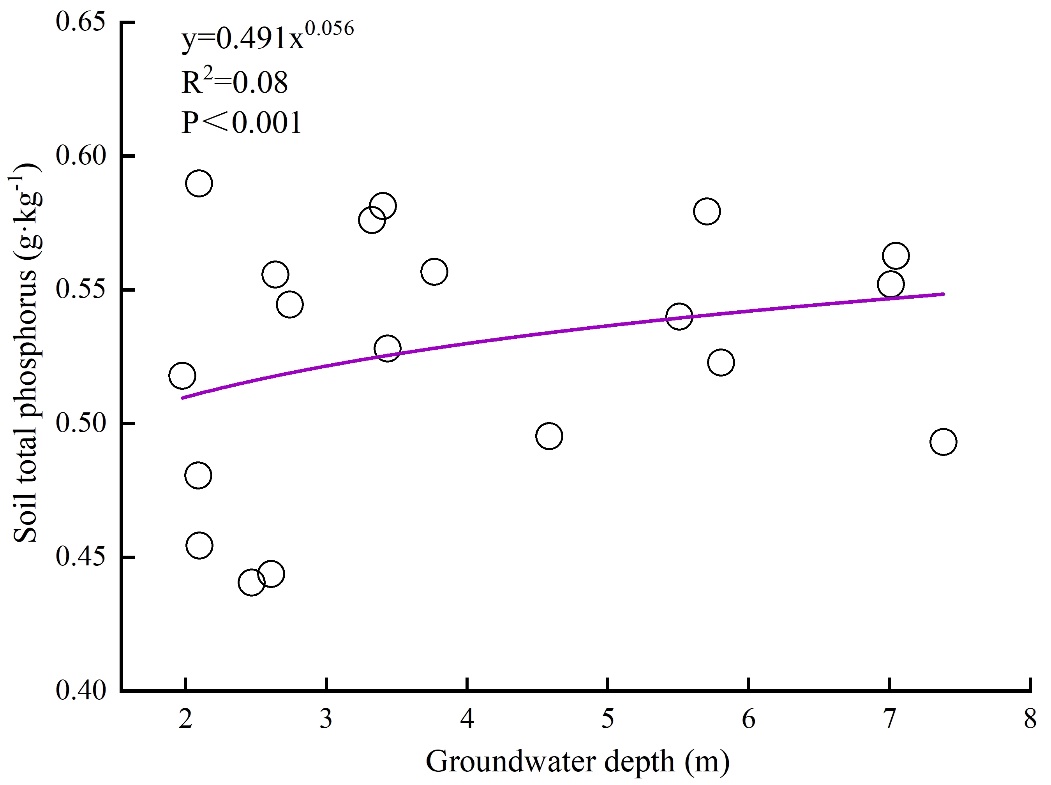
S****upplementary Figure 1.** Response pattern of soil total phosphorus to groundwater depth

**
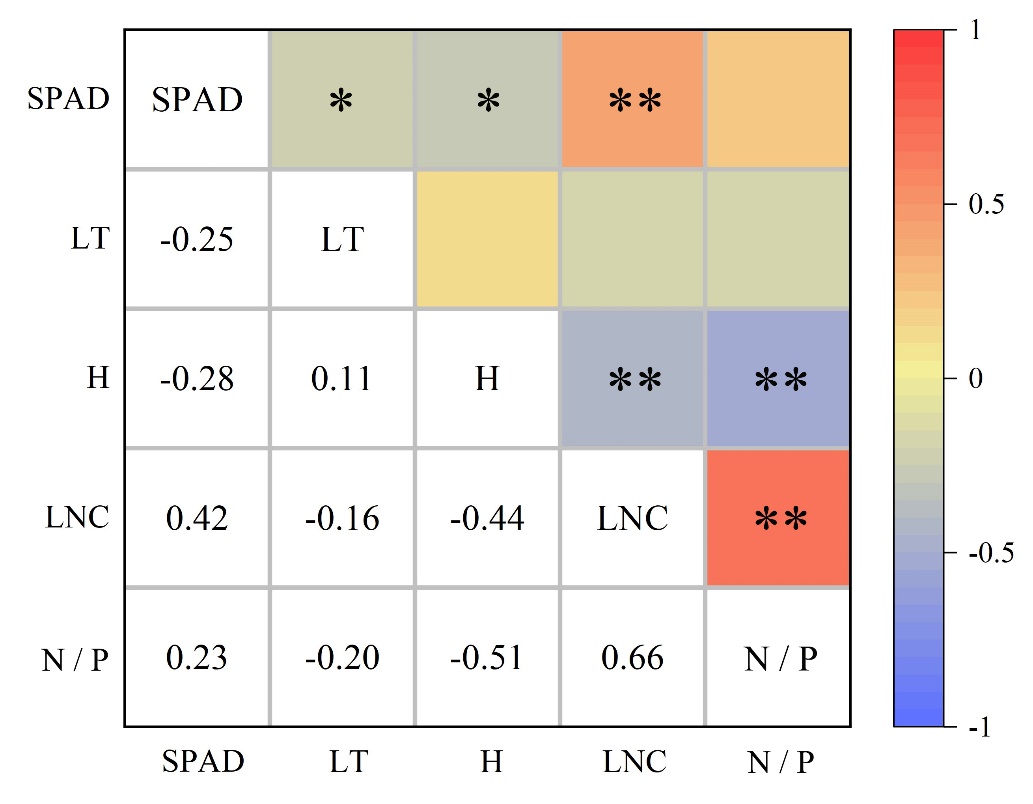
****Supplementary Figure 2.** Results of Spearman's correlation analysis between community weighted mean traits

# Further notes on the vegetation survey

Since species were sparse in most of the plots and herbaceous plants grew singly, every plant within the plot was measured. However, there is a special situation in measuring *Phragmites australis* in plots 1, 3 & 16, which have high density and uniform distribution of *P*. *australis*. We randomly selected five small herbaceous subplots of 1 × 1 m within each subplot for *P*. *australis* surveys and used them to estimate indicators such as the number of *P*. *australis* within the subplot (Supplementary Figure 3).


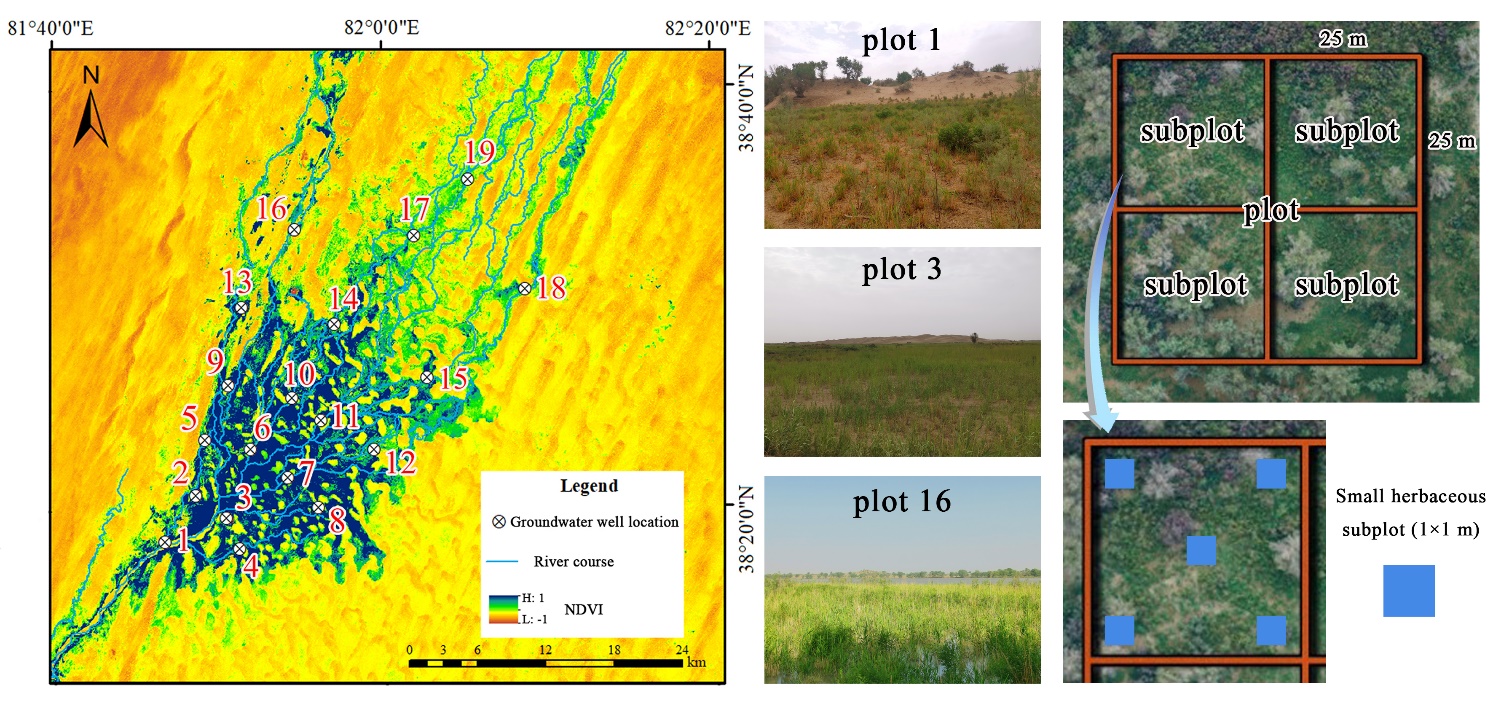
**Supplementary Figure 3.** *Phragmites australis* survey methods for plots 1, 3, and 16
